# Novel Perspective on the Plasticity of Taste Perception: Is Food- and Exercise-Induced Inflammation Associated with Sweet Taste Sensitivity and Preference?

**DOI:** 10.1021/acs.jafc.3c09028

**Published:** 2024-06-28

**Authors:** Isabella Kimmeswenger, Barbara Lieder

**Affiliations:** † Department of Physiological Chemistry, Faculty of Chemistry, University of Vienna, Josef-Holaubek-Platz 2, 1090 Vienna, Austria; ‡ Vienna Doctoral School in Chemistry (DoSChem), University of Vienna, 1090 Vienna, Austria; § Christian Doppler Laboratory for Taste Research, Faculty of Chemistry, University of Vienna, Josef-Holaubek-Platz 2, 1090 Vienna, Austria; ∥ Institute of Clinical Nutrition, University of Hohenheim, 70599 Stuttgart, Germany

**Keywords:** taste perception, taste preferences, inflammation, food choices, physical activity

## Abstract

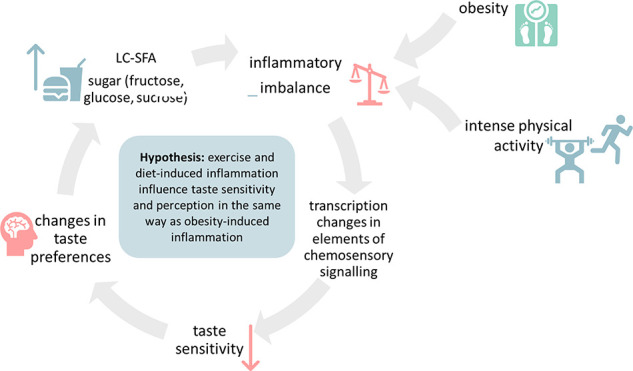

Obesity-related inflammation has been linked to decreased taste sensitivity and changes in the transcriptome of the taste apparatus. Increased levels of pro-inflammatory cytokines can also be found to be food-associated in individuals who consume high amounts of long-chain saturated fatty acids and sucrose independent of the body composition or individuals who exercise intensively. Previous research suggests a link between taste sensitivity and food choices. However, the interplay between food- or exercise-induced low-grade inflammation, taste perception, and food choices remains unaddressed. Understanding this relationship could provide an unnoticed explanation for interindividual differences in taste perception that influences dietary habits.

## Introduction

Establishing healthy food choices is of great importance, because it can decrease the risk for the development of diseases. However, adhering to nutritional recommendations can be challenging, as dietary behavior is very complex and influenced by a variety of factors. Besides cultural, social, and psychological influences, physiological signals as well as sensory signals are described as major driving forces for food choices.^1^ Parts of the sensory signal apparatus can be influenced by food components. Therefore, food choices and with that the uptake of certain nutritive or anutritive compounds and the sensory signaling may influence each other.

The influence of flavor on dietary behavior can be explained by the fact that its perception provides the human organism with the last information about the quality and chemical composition of the food before ingestion.^2^ This mechanism makes particular sense from an evolutionary perspective, although the extent to which sensory attributes influence food choices is controversial. Kourouniotis et al. showed that 82% of the study population considered taste to be an important factor in food selection.^3^ Contrasting results were found in a study with 589 adults of Indian origin, in which only 20% of the participants considered taste to be the primary factor influencing their food choices,^4^ indicating that there might be regional differences, influenced by an interplay of cultural, economic, or social factors.

One major part of the sensory signals and with that flavor perception is transmitted via taste receptors. According to the current state of knowledge, five taste qualities are scientifically accepted. These are sweet, sour, bitter, salty, and umami. Results from animal and human studies additionally suggest the existence of further taste modalities, among them being fatty, kokumi, or the ability to detect essential nutrients, such as Ca^2+^.^2^

Taste perception starts at the level of oral taste receptor cells. These group themselves up to onion-shaped taste buds, which can be found in different kinds of papillae, localized in tongue epithelium and mucosa of the oral cavity. One taste bud consists of 50–100 sensory cells, which can be divided into three main cell types, according to their function.^2^ Type I cells account for half of the cells in the taste bud. Their functions seem to resemble those of glial cells. Type II cells possess chemosensory receptors for sweet, bitter, and umami stimuli. They express specific types of G-protein-coupled receptors, which is why each cell can only detect one taste quality, whereas one taste bud can detect several taste stimuli, as it consists of different types of type II cells.^2^ Type III cells detect sour stimuli. Unlike type II cells, their taste detection is not based on G-protein-coupled receptors but on proton-selective ion channels, encoded by otopetrin 1 (OTOP-1).^5^ Only little is known about the perception of salty taste. In contrast to rodents, salt perception in humans is insensitive to amiloride, for which cells of types II and III have been implicated to be responsible.^6,7^ Most recent results from Roebber et al. indicate that more than 80% of NaCl-reactive taste bud cells could be assigned to cells of type II when using confocal Ca^2+^ imaging via GCaMP3 expressed in types II and III taste bud cells. In addition, the data suggested that rather the chloride anion and not the sodium ion is mediating the amiloride-insensitive salt taste.^8^

The expression profile of taste cells and receptors is crucial for taste perception, and the regulation can be influenced by the interaction with specific tastants through the diet. For example, studies in mice show that a four week exposure to either 30 mM monosodium glutamate, 2 mM saccharin, or 90 mM NaCl as prototypical taste stimuli for umami, sweet, and salty taste was associated with a significant decrease in the mRNA expression for respective umami, sweet, and salty receptors/channels.^9^ Previous research focused on further influencing factors; however, there are still a lot of interindividual differences in taste perception that cannot be explained by the already known ones. Thus, this perspective briefly discusses already established influencing factors and their effects and presents a new hypothesis regarding a potential influencing factor that has received only little attention to date.

## Differences in Taste Perception Can Be Linked to Age, Sex, and Obesity

It is well-established that there are interindividual differences in taste perception, but the underlying reasons for these differences remain partly unclear. Possible causes may include physiological variations in the gustatory system (such as differential expression patterns of taste receptors), cognitive processing of taste signals in the brain, genetics, or environmental factors.^10^ While little is known about the exact cause of varying taste perception, numerous studies associated taste perception with lifestyle factors. Frequently discussed factors include age, sex, and obesity.^10^ In particular, the increase in age is a well-described important factor in the declining sensitivity to taste stimuli, and Barragan et al. demonstrated that, with advancing age, intensity ratings for all taste qualities decrease.^11^ Furthermore, sex differences were previously discussed. A study with 1020 participants showed significantly higher intensity ratings in female compared to male participants for sour, bitter, and salty stimuli.^11^ Results from Pulputti et al. also demonstrated that females are more sensitive toward taste stimuli by having on average a 0.24 unit higher taste sensitivity score than men, corresponding to approximately 13%.^10^ A possible cause of the difference in taste perception could be differences in the gustatory system as a result of sex hormones, such as estrogen.^12^ While the factors of sex and age cannot be influenced individually, at least partly modifiable lifestyle factors have also become the focus of research in recent years.

## Obesity Influences Sensitivity toward Taste Stimuli

One factor that has been extensively studied to influence taste sensitivity is obesity. However, the literature on this topic is controversial, as there are findings that indicate that obese individuals exhibit higher, lower, or unchanged taste sensitivities compared to normal-weight subjects.^13−15^ In more detail, Hardikar et al. demonstrated 49% lower recognition thresholds to sweet and 52% lower recognition thresholds to salty stimuli in their obese study population compared to a lean control group,^13^ indicating a higher sensitivity in obese test persons. On the other hand, Low et al. found no association between sweet sensitivity and the anthropometric characteristics of waist circumference and body mass index (BMI).^14^ In contrast, Prosperio et al. demonstrated significantly higher detection thresholds for sweet, salty, bitter, fatty, and sour taste stimuli alongside a significantly lower fungiform papillae density in obese subjects compared to a normal-weight control group.^15^

## Obesity-Related Changes in Taste Perception Might Be Linked to Inflammatory Dysbalances

As a possible cause for reduced taste sensitivity in obese individuals, obesity-induced inflammation is discussed. Kaufman et al. showed that mice had on average 40.9 ± 5.7 fewer taste buds per circumvallate papillae and a 45-fold-increased relative gene expression of tumor necrosis factor α (TNF-α) in the circumvallate papillae after eight weeks of a high-fat diet compared to wild-type mice fed with standard chow. They suggested that inflammation leads to morphological changes that subsequently impair taste perception.^16^ In a follow-up study, the authors found a negative correlation between the fungiform papillae density and body weight in mice (*r* = −0.78). Similarly, a negative correlation between the fungiform papillae density and neck circumference, which was used as an indicator of obesity, was observed in a human population (*r* = −0.37).^17^ These findings further support the results of a meta-analysis conducted by Trius-Soler et al., who showed that a higher BMI is associated with a higher detection threshold for sweet taste. Interestingly, this overall effect is not only based on comparing a normal-weight group to an overweight group but also the combination with studies that examined taste perception in obese participants before and after weight loss.^18^ These results could indicate that reduced taste sensitivity in obese participants is due to an increase in inflammatory parameters, as studies have shown that both invasive and non-invasive weight loss is associated with reduced concentrations of the pro-inflammatory cytokine C-reactive protein (CRP).^19,20^ However, it has to be noted that results of cross-sectional studies with overweight or obese subjects may be biased as a result of differences in the underlying pathogenesis and should, therefore, be interpreted with caution.

## Inflammation as a Key Driver for Sensory Deviations?

Non-obesity-associated inflammation appears to affect taste perception, as it became particularly evident in recent studies related to coronavirus disease 2019 (COVID-19). COVID-19 patients with xerostomia and taste loss showed 28% increased concentrations of the pro-inflammatory cytokine interleukin 8 (IL-8) in serum compared to a group without taste loss.^21^ However, it has to be noted that changes in taste also often occur in cancer-related xerostomia, as summarized by Galaniha and Nolden.^22^ Also, other inflammatory diseases are linked to changes in the perception of sensory signals. For example, patients with rheumatoid arthritis showed a reduced chemosensory function.^23^ In inflammatory bowel disease patients, a progressed stage of the disease was associated with a reduction in olfactory sensitivity, whereby a subgroup analysis also revealed that a four month treatment with TNF-α inhibitors led to an improvement by 6% in olfactory function, measured by a score consisting of sensitivity, discrimination, and identification.^24^

In addition, inflammation could also be the underlying cause of altered taste perception when taking various chemotherapeutic agents. For example, the drug cisplatin inhibits proliferation and increases apoptosis rates as well as the concentrations of inflammatory cytokines and chemokines TNF-α, interleukin 9 (IL-9), interleukin 12 (IL-12), monocyte chemoattractant protein 1a (MCP-1a), eotaxin, granulocyte colony-stimulating factor (G-CSF), and interferon γ (IFN-γ) in cells of the circumvallate papillae in mice, leading to impairments in taste function. These findings were supported by studies conducted in a murine taste bud organoid model.^25^

Inflammatory processes can be also influenced by food components.^26^ For example, the excessive dietary intake of long-chain saturated fatty acids (LC-SFAs) and sugar, especially fructose, is associated with pro-inflammatory processes.^27,28^ In more detail, Berg et al. showed a correlation between the daily consumption of 29 ± 13 g of LC-SFAs with an increase in the kynureine:tryptophan ratio, a marker for inflammation.^24^ Similarly, Cox et al. showed an increase of 84 ± 33% for the concentrations of MCP-1 and an increase of 29 ± 14% for E-selectin when 25% of the energy requirement was covered by fructose in the form of a sweetened soft drink. In contrast, no increase in inflammatory factors was observed when the same intervention was carried out with glucose.^25^

An increased intake of the aforementioned pro-inflammatory food components is also associated with changes in taste perception. This effect is independent of weight gain or associated obesity.^29,30^ Mice on a high-fat diet, providing 60% of the energy from fat, for six to eight weeks had significantly reduced expressions of genes encoding for α-gustducin and phospholipase Cβ2, two key signaling proteins of the chemosensory signaling transduction independent of weight gain.^29^ Furthermore, mice fed a typical Western diet, providing 39% fat over 24 weeks, with additional consumption of a beverage sweetened with 11.2% glucose–fructose syrup showed a 36% reduced chemosensory surface from fungiform papillae compared to mice fed the same diet but receiving water instead of the sugar-sweetened beverage.^30^ However, the current studies do not allow for differentiation between the effects of various short-chain carbohydrates on the chemosensory signaling. Although there is an association between nutrients that are thought to have a pro-inflammatory effect and markers of taste perception, there is a lack of studies that systematically investigated the relationship between these nutrients, taste perception, and inflammatory processes. Subsequently, the question arises of whether anti-inflammatory food components have an effect on the regulation of taste perception. Substances that could be of interest in this context are ω-3 fatty acids or polyphenols.^26^ In this regard, it may also be of interest to investigate whether these anti-inflammatory substances could counteract an inflammation-induced impairment of taste perception.

## Intensive Physical Activity Provokes a Rise in Inflammatory Cytokines

Food and dietary choices are an important factor influencing inflammatory processes, and a factor that can be regulated by the lifestyle of an individual.^26^ However, the extent to which nutrient and non-nutrient associated pro- or anti-inflammatory effects influence taste sensitivity and preferences still requires further research. Another lifestyle factor with inflammatory potential that should not be neglected is exercise.

Although low-grade inflammation is a condition often associated with overweight, obesity, and physical inactivity,^31^ increased cytokine levels are also found, especially in individuals who exercise intensively. For example, fasting concentrations of interleukin 6 (IL-6), interleukin 8 (IL-8), and interleukin 15 (IL-15) are significantly higher in elite strength athletes compared to untrained healthy subjects.^32^ Similarly, in elite runners, baseline concentrations of plasma malondialdehyde (MDA), a marker for oxidative stress, were 87% higher compared to inactive controls.^33^ Notably, the mentioned study groups consisted of athletes whose level of physical activity is significantly higher than that of the average population.

While a number of studies examined the relationship between exercise and inflammation as well as the interaction between taste perception and obesity-driven inflammation, the interplay between exercise-induced inflammation and taste perception has not yet received attention. Results from our research group as well as the study by Iatridi et al. demonstrate a connection between sweet perception, preference, and physical activity,^34,35^ and preliminary, unpublished results of our group additionally indicate that the underlying mechanism of this interaction could be attributed to inflammatory processes involving IL-6.

## Inflammation Arising from Physical Activity as a Potential Regulator of Sweet Taste Perception and Preferences

In the course of a previous human study of our own group, the sweet perception, hedonic preference for sweet-tasting foods, and body composition of subjects were determined, among others.^35^ The study population was young, metabolically healthy male subjects, with BMI of 18.5–29.99 kg/m^2^ and body fat percentage of <30%. Results of this study show that the detection threshold for sweet taste in almost half of the subjects is above that range that is achieved in a comparable population according to DIN ISO 3972:2013-12. Subjects with a sweetness threshold above 4.32 g/L showed a lower body fat percentage (13 ± 7%) than the subjects with a threshold up to 4.32 g/L (17 ± 9%). It is likely that the differences in body fat are related to the physical activity level of the participant.^35^ We hypothesize that the underlying cause is based on increased levels of pro-inflammatory cytokines, such as IL-6, which are known to be elevated in frequent intense physical activity.^32^ This inflammatory imbalance could lead to similar effects as observed by Kaufman et al., who found increased apoptosis rates in taste cells with obesity-induced inflammation in mice.^16^ We suggest that intense physical activity can lead to elevated cytokine levels, which may subsequently influence taste perception and preference. High levels of intense physical activity result in an increased energy requirement. The observation of higher liking for sweet foods in physically very active subjects could be explained by the presence of an inflammation-mediated energy signaling, which regulates the preference for rapidly available nutrients to satisfy energy needs.

## Outlook and Concluding Remarks

Understanding how different taste sensitivities arise can help to clarify differences in food preferences. Subsequently, research on the underlying mechanisms can help to implement healthy dietary choices in a more targeted way. It is already known that obesity-related low-grade inflammation can reduce taste sensitivity.^16^ We hypothesize that pro-inflammatory food components, such as LC-SFAs and short-chain carbohydrates, as well as intense physical activity similarly lead to such an inflammatory imbalance, which, in turn, influences taste perception. In more detail, we would expect a rise in pro-inflammatory cytokines, such as different interleukins, leading to elevated apoptosis rates of taste cells in the oral cavity. This would result in a decreased number of chemosensory receptors detecting taste stimuli and forwarding signals to the brain, leading to potentially decreased sensory perception. Furthermore, it could be possible that elevations in pro-inflammatory mediator levels affect gustatory nerves or influence transcription in chemosensory signaling.

Exercise-enhanced inflammation is the result of leukocyte mobilization and release of cytokines from activated muscle tissue induced by a stressor in the form of high-intensity physical activity.^36^

The mentioned food components activate inflammatory pathways differently. Consumption of LC-SFAs can lead to a concentration rise in IFN-γ, which upregulates indoleamine 2,3-dioxygenase (IDO), resulting in a reduced T-cell activation and proliferation.^27^ There are several ways in which mono- and disaccharides influence inflammation.^26^ The monosaccharide fructose on one side might stimulate an immune response by activating protein kinase K in the liver, enhancing MCP-1 production.^28^ Diets with a high glycemic index on the other side have the potential to disturb gut homeostasis, favoring the circulation of lipopolysaccharides.^26^

**Figure 1:**
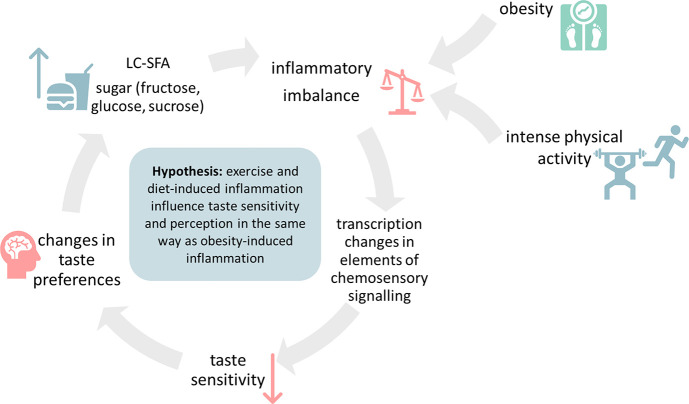
Graphical overview of the hypothesis. We hypothesize that intense physical activity affects taste perception in a manner similar to obesity and the consumption of a pro-inflammatory diet. Accordingly, both exercise- and obesity-induced inflammation lead to a decrease in taste sensitivity and, as a result, influence taste preferences.

Inflammation-mediated changes in taste perception may result in changes in taste preference, which, in turn, can shape dietary habits and food choices (Figure 1). This could be particularly relevant for athletes who show a reduced taste sensitivity and high liking for sweet-tasting substances and, therefore, consume amounts of foods and beverages rich in sugar, exceeding the recommendations of the World Health Organization. The consumption of high amounts of sucrose and its derivatives glucose and fructose not only increases pro-inflammatory cytokines but also favors blood sugar fluctuations and can result in further cravings for sweet. This can for instance happen as a result of hypoglycemic blood sugar levels, which could have been observed in non-diabetic participants after consumption of a 250 mL drink containing 80 g of sucrose. The results may be a vicious circle, which, in the long run, can lead to metabolic diseases caused by excessive sugar consumption.^37^

However, to confirm these relationships, studies in larger cohorts are needed that examine the target groups of interest and exclude the limitations mentioned above. In addition to the influence of age, sex, obesity, and pathologically or pharmacologically induced inflammation, the effect of exercise-induced inflammation on taste sensitivities as well as possible regulatory functions of food components are aspects that have not yet been addressed. Such results could provide a thus far unnoticed explanation for interindividual differences in taste perception and simultaneously help to promote healthy food choices by deriving personalized advice.

## Abbreviations Used

BMI: body mass index
COVID-19: coronavirus disease 2019
G-CSF: granulocyte colony-stimulating factor
IDO: indoleamine 2,3-dioxygenase
IFN-γ: interferon γ
IL-6/8/9/12/15: interleukin 6/8/9/12/15
LC-SFAs: long-chain saturated fatty acids
MDA: malondialdehyde
MCP-1: monocyte chemoattractant protein 1
OTOP: otopetrin
TNF-α: tumor necrosis factor α
